# Educational Case: Molecular basis of follicular thyroid nodules

**DOI:** 10.1016/j.acpath.2026.100292

**Published:** 2026-08-14

**Authors:** Abdulla Kamashki, Priya Upadhyay, Bidhisa Kharel, Swikrity Upadhyay Baskota

**Affiliations:** a Department of Pathology and Laboratory Medicine, Albany Medical Center, Albany, NY, USA; b Department of Pathology and Laboratory Medicine, UC Davis Medical Center, Sacramento, CA, USA; c Department of Internal Medicine, Presence St. Francis, Evanston, IL, USA

**Keywords:** Pathology competencies, Organ system pathology, Thyroid neoplasms, Thyroid nodules, Endocrine, Endocrine neoplasms, Fine needle aspiration, Molecular testing

The following fictional case is intended as a learning tool within the Pathology Competencies for Medical Education (PCME), a set of national standards for teaching pathology. These are divided into three basic competencies: Disease Mechanisms and Processes, Organ System Pathology, and Diagnostic Medicine and Therapeutic Pathology. For additional information, and a full list of learning objectives for all three competencies, see https://doi.org/10.1016/j.acpath.2023.100086.^1^

## Primary objective

Objective EN5.1: Thyroid Neoplasms. Compare and contrast the clinicopathologic features, pathogenesis, and prognosis of follicular adenomas, follicular carcinoma, and papillary thyroid carcinoma.

Competency 2: Organ System Pathology; Topic: Endocrine (EN); Learning Goal 5: Endocrine Neoplasms.

## Patient presentation

A 61-year-old woman presents to her family physician with a concern of swelling on the left side of her neck. Her swelling was not associated with fatigue, weight changes, cold or heat intolerance, mood or sleep disturbances, weakness, palpitations or gastrointestinal issues. Her past medical history is noncontributory. She has no history of radiation exposure and no family history of thyroid cancer.

## Diagnostic findings, Part 1

On physical examination, a mobile, nontender nodule of approximately 1 cm in size palpated in the region of the left lobe of the thyroid gland. The nodule moves on swallowing. The patient appears clinically euthyroid.

## Questions/discussion points, Part 1

### What are the differential diagnoses for a patient presenting with a thyroid nodule?

More than 90% of palpable/incidentally detected thyroid nodules are benign follicular nodular disease.^2^ This category includes multinodular goiter, colloid nodules and adenomatoid hyperplasia, which typically occur in clinically euthyroid patients. Other entities in the differential diagnoses include inflammatory or autoimmune conditions such as granulomatous thyroiditis, Hashimoto's thyroiditis, lymphocytic thyroiditis, viral thyroiditis and Graves' disease; however, these usually present with abnormal thyroid hormone levels.

## Diagnostic findings, Part 2

Laboratory investigation shows a TSH (Thyroid Stimulating Hormone) level of 4.02 mIU/L (reference range = 0.7–5.07 mIU/L) and a free T4 level of 0.95 ng/dl (reference range = 0.62–1.15 ng/dl), both within normal limits. These findings are consistent with a nonfunctioning thyroid nodule, which may be either benign or malignant.

For patients presenting with a thyroid nodule and low serum TSH, an ultrasound (US) is performed to evaluate the nodule and assess for features suggestive of thyroiditis. If a hyperfunctioning nodule is suspected, a radionucleotide thyroid scan is used to evaluate the functional status of the nodule. This helps in differentiating a hyperfunctioning nodule, which is rarely malignant, from a nonfunctioning nodule, which requires further cytological or histological evaluation.

Conversely, the presence of a thyroid nodule with elevated or borderline high TSH levels may also be associated with a higher risk of malignancy (ROM) and warrants further evaluation.^3^

A minority of the palpable or incidental thyroid nodules are neoplastic, which can be confirmed by histomorphology and/or the presence of clonal neoplastic mutations. Among these, only a small proportion (approximately 4–6.5%) are malignant.^2^ Additional structures mimicking thyroid nodules may include an intrathyroidal parathyroid glands, parathyroid adenomas,^4^ or intrathyroidal lymph nodes.^5^ A summary of the differential diagnosis for thyroid nodules is provided in Table 1.

**Table 1:** Differential diagnosis, clinical, and cytologic features of a thyroid nodule.

Group	Diagnoses	Thyroid hormone	Follicular cells	Colloid
Benign follicular nodular disease	Colloid nodule adenomatoid hyperplasia	Typically euthyroid	Monolayered sheets, three dimensional clusters	Some or abundant
Benign	Graves disease	Typically hyperthyroid, can be euthyroid	Cellular sheets of benign follicular cells	Scant or absent
Benign	Granulomatous thyroiditis, lymphocytic or infectious thyroiditis	Euthyroid or hypothyroid	Granulomata, scant follicular cells, lymphocytic tangles	Scant
Neoplastic	Follicular or oncocytic adenoma	Euthyroid	Predominantly microfollicles or >90% of oncocytes	Scant or absent
Neoplastic	Noninvasive follicular thyroid neoplasms with papillary like nuclear features (NIFTP)	Euthyroid	Predominantly microfollicles with overlapping crowding, pale nuclear chromatin, margination of nucleoli, with or without intranuclear inclusions, no definitive papillae	Scant or absent
Malignant	Papillary thyroid carcinoma	Euthyroid	Cellular, with papillae with fibrovascular core, psammoma bodies, optically clear nuclei, intranuclear inclusions and grooves	Scant or absent
Malignant	Follicular carcinoma	Euthyroid	Similar to follicular adenoma	Scant or absent
Malignant	Medullary thyroid carcinoma	Euthyroid, calcitonin high	Plasmacytoid, discohesive para-follicular cells, sometimes in microfollicles with anisonucleosis, with or without amyloid	Absent
Malignant	Undifferentiated/poorly differentiated thyroid carcinoma	Euthyroid or borderline high TSH	Pleomorphic malignant cells, with necrosis, mitoses, anaplasia	Scant or absent
Malignant-rare	Primary thyroid lymphoma or metastatic disease	Euthyroid or borderline high TSH^1^	Nonthyroidal malignant lymphoid cells or other epithelial cells	Can be present
Extrathyroidal	Intrathyroid parathyroid or parathyroid adenoma	Euthyroid, serum PTH can be high	Microfollicular arrangement of monotonous appearing parathyroidal cells often interpreted as follicular cells	Can be present

## Questions/discussion points, Part 2

### How can these lab findings direct further investigations?

The normal lab values indicate that the patient is euthyroid. The thyroid nodule can be either benign or malignant, and US should be performed for further evaluation.

## Diagnostic findings Part 3

Thyroid US revealed a hypoechoic nodule with punctate echogenic foci in the left lobe of the thyroid gland, measuring 12 × 9 × 8 mm. The nodule demonstrated irregular margins.

## Questions/discussion points, Part 3

### What are the indications for a FFNA of the thyroid?

Fine needle aspiration (FNA) is the current standard of care for evaluating the ROM of thyroid nodules and determining the need for surgical intervention. The decision to perform an FNA is primarily guided by the US features of the nodule, along with relevant clinical information.

The thyroid imaging reporting and data systems (TIRADS) are used to assess and stratify the ROM based on specific US characteristics: composition, echogenicity, shape, margins, and echogenic foci. Based on the cumulative score, nodules are classified into five categories: TIRADS 1 as benign, TIRADS 2 as not suspicious, TIRADS 3 as mildly suspicious, TIRADS 4 as moderately suspicious, and TIRADS 5 as highly suspicious. The risk category, along with the nodule size, determines whether FNA is indicated or if the nodule can be monitored with repeated US^6^ (Table 2).

**Table 2:** Risk stratification suggested management of thyroid nodules based on the TIRADS categories.

Total score	0	2	3	4–6	≥7
**Risk category**	TR1 (Benign)	TR2 (not Suspicious)	TR3 (mildly suspicious)	TR4 (moderately suspicious)	TR5 (highly Suspicious)
**Management**	No FNA	No FNA	FNA if ≥ 2.5 cm Follow if ≥ 1.5 cm	FNA if ≥ 1.5 cm Follow if ≥ 1 cm	FNA if ≥ 1 cm Follow if ≥ 0.5 cm

FNA: fine needle aspiration.

## Diagnostic findings, Part 4

The patient underwent an US-guided FNA of the nodule following its classification as **TIRADS 5** on US, indicating high suspicion for malignancy.

## Questions/discussion points, Part 4

### How is thyroid FNA cytology reported?

With the goal of standardizing the reporting of thyroid FNA cytology, The Bethesda System for Reporting Thyroid Cytopathology (TBSRTC) was introduced in 2010, and most recently updated in 2023. The updated system classifies thyroid lesions into six diagnostic categories: I (nondiagnostic), II (benign), III (atypia of undetermined significance [AUS]), IV (follicular neoplasm [FN]), V (suspicious for malignancy [SFM]), and VI (Malignant). TBSRTC plays a key role in risk stratification, as each category is associated with an estimated ROM and corresponding management recommendations. This system helps guide clinicians in making informed decisions regarding the evaluation and treatment of thyroid nodules^7^ (Table 3).

**Table 3:** Clinical management algorithm as suggested by TBSRTC 2023.

Diagnostic category	Nondiagnostic	Benign	AUS	FN	SFM	Malignant
**ROM % (mean)**	13 (5–20)	4 (2–7)	22 (13–30)	30 (23–34)	74 (67–83)	97 (97–100)
**Management**	Repeat FNA with ultrasound-guidance	Follow-up clinically and with ultrasound	Repeat FNA, lobectomy, molecular testing, or surveillance	Molecular testing, lobectomy	Molecular testing, near-total thyroidectomy, or lobectomy	Near-total thyroidectomy or lobectomy

TBSRTC: The Bethesda System for Reporting Thyroid Cytology; AUS: atypia of undetermined significance; FN: follicular neoplasm; SFM: suspicious for malignancy; ROM: risk of malignancy; FNA: fine needle aspiration.

## Diagnostic findings, Part 5

**Fig. 1:**
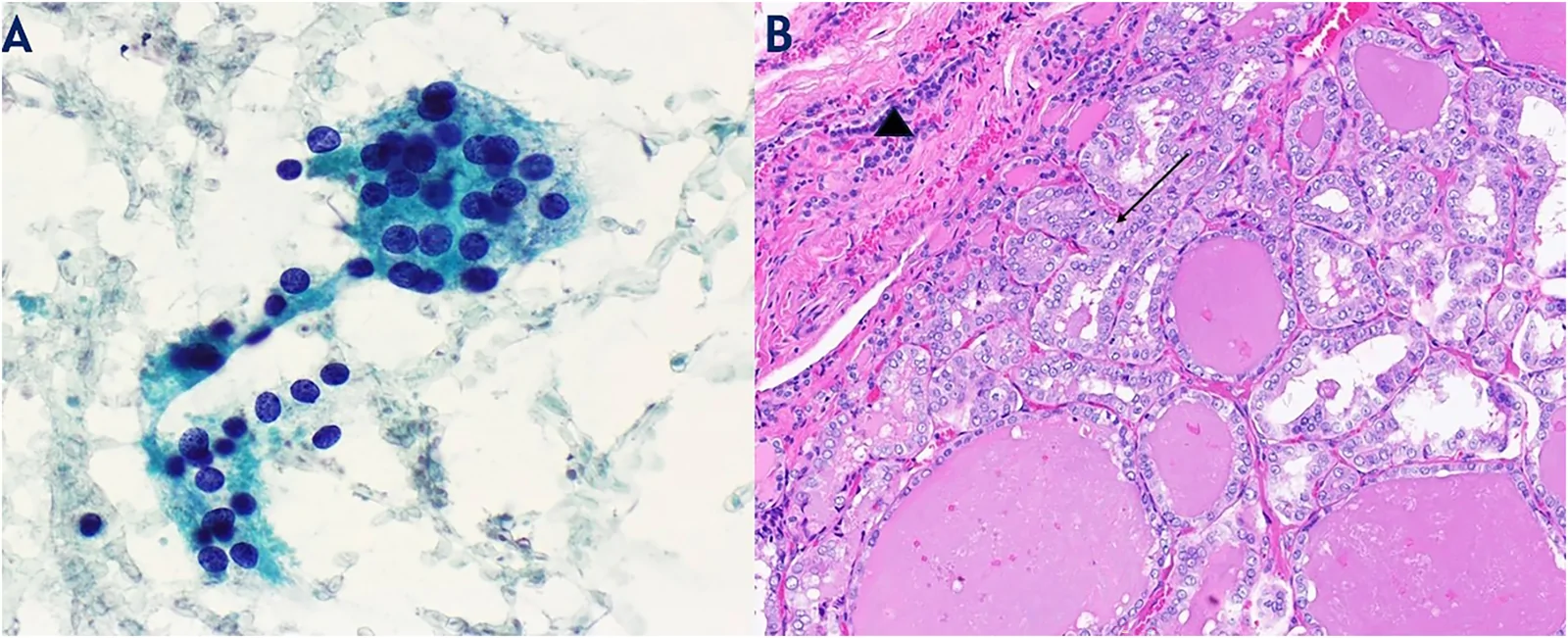
Cytology and subsequent resection diagnosis of the thyroid nodule of our case. **(A)** Rare sheets and a loose microfollicular arrangement of atypical thyroid follicular cells with scant colloid in the background, which was reported to have a KRAS G12A mutation by molecular analysis (Thyroseq, V3) (ThinPrep, 20X, Papanicolaou stain). **(B)** Subsequent thyroidectomy revealed a noninvasive follicular thyroid neoplasm with papillary-like nuclear features (NIFTP). Neoplastic cells exhibit nuclear clearing, pseudoinclusions and microfollicular architecture (black arrow). Normal thyroid tissue is also shown (black arrow head). (FFPE, 20X, H&E stain).

The thyroid FNA cytology was reported as FN, TBSRTC category- IV. [Fig. 1].

## Questions/discussion points, Part 5

### What are the cytomorphological features useful for the diagnosis of a follicular neoplasm?

Category-IV of the TBSRTC refers to FNs. Aspirates from FNs are typically cellular, consisting predominantly of mildly atypical follicular cells arranged in a microfollicular architecture. Cytologically, microfollicles are defined as flat groups of less than 15 follicular cells with nuclear crowding and overlapping.^7^

Although the background colloid is usually scant, some cases may demonstrate abundant colloid. The presence or absence of pigment-laden macrophages or blood is not diagnostically significant in this category. The atypical follicular cells in these aspirates lack the classic nuclear features of papillary thyroid carcinoma (PTC) (e.g. nuclear grooves, inclusions, and clearing) as described in Table 1.

Oncocytic neoplasms, a subset within FN are identified when the aspirate predominantly contains oncocytic cells. These cells display abundant granular cytoplasm, moderate anisonucleosis, prominent nucleoli, and scant colloid. A helpful cytologic feature supporting the diagnosis of oncocytic neoplasms is the presence of traversing blood vessels within the cell groups.

It is important to note that the cytology alone cannot distinguish between follicular adenoma and follicular carcinoma, as the diagnosis requires histologic evidence of capsular and/or vascular invasion. Approximately (30%) of FNs are diagnosed as follicular/oncocytic carcinoma upon surgical resection. Because of this limitation, the cytologic term “FN” is used broadly to encompass both follicular adenomas and carcinomas.

### What is the best next step in the evaluation of indeterminate thyroid nodules?

TBSRTC categories III-V are considered indeterminate thyroid nodules. In these categories, cytology alone is insufficient to differentiate between benign and malignant lesions. Therefore, repeat biopsy (category III), molecular testing or surgery, based on the patient's preference play a crucial role in guiding further management. The ROM for category III and IV is 22% and 30%, respectively (Table 3). For Bethesda category III, the recommended next steps include either repeat FNA or reflex molecular testing using thyroid-specific mutation panels. For Bethesda category IV (FN), the management options include either molecular testing or lobectomy depending on clinical and sonographic risk factors. For category V (suspicious for malignancy/SFM), the suggested management is lobectomy, with or without the use of molecular testing.

Because these indeterminate categories encompass a heterogeneous group of lesions with overlapping cytologic and architectural features, adjunct molecular testing is being used more frequently to improve diagnostic accuracy. Molecular testing can enhance risk stratification by identifying specific genetic mutations or rearrangements, helping to better characterize nodules and reduce unnecessary or extensive surgeries (i.e. avoiding total thyroidectomy when lobectomy would suffice^8^^,^^9^).

## Diagnostic findings, Part 6

The FNA cytology was reflexed for ThyGeNEXT/ThyraMIR molecular panel testing. ThyGeNEXT showed an NRAS mutation at an allelic frequency of 35% with a reported associated ROM of ∼50%.

## Questions/discussion points, Part 6

### What are the common molecular alterations in follicular-patterned thyroid nodules?

Follicular-patterned thyroid neoplasms are primarily driven by activation of the PI3K/AKT signaling pathway. This pathway is typically activated either by activating mutations in *PIK3CA*, *RAS (NRAS, HRAS, and KRAS)* and *AKT1,* or by inactivation of *PTEN*, a tumor suppressor gene that normally inhibits this pathway.^10^

Additional molecular changes, such as mutations in *p53*, *Wnt/B-catenin*, and the *TERT* promoter, are implicated in the dedifferentiation of well-differentiated thyroid carcinoma into more aggressive undifferentiated forms.^11^

A summary of the most common molecular alterations and their characteristics in follicular-patterned thyroid lesions is described in Table 4.

**Table 4:** Common genetic alterations in follicular-patterned thyroid neoplasms.

Diagnosis	Mutation	Frequency
Follicular adenoma	RAS-point mutation	20–40%
	PAX8-PPARγ translocation	5–20%
	Germline PTEN mutation in association with Cowden syndrome	Rare
Follicular thyroid carcinoma^a^	RAS- point mutation	30–50%
	PAX8-PPARγ translocation	30–35%
	TERT	10–20%
	PTEN	<10%
	PIK3CA	5–10%
Oncocytic adenoma	Copy number variations	
	RAS-point mutations	
Oncocytic variant of folliicular thyroid carcinoma	Alterations in mitochondrial DNA including deletions, frameshift, and missense mutations	
	GRIM19 mutation	10–20%
	RAS	10–20%
	PAX8-PPARγ translocation	5–15%
	TERT	15–20%
	TP53	Upto 20%
	RET/PTC translocations with solid growth pattern	35% (based on one study)
NIFTP	RAS	40–45%
	PAX8-PPARγ translocation	Upto 20%
	THADA fusion	Upto 20%
	EIF1AX	5%
	Absence of BRAF mutations and RET/PTC translocations	
Follicular variant of papillary thyroid carcinoma	RAS	25–50%
	PAX8-PPARγ translocation	5–30%
	BRAFv600E^b^	Upto 25% (invasive type)
	TERT	1–10%
	RET/PTC translocations^b^	5%
	NTRK	0–10%
	BRAF K600E	<1%

a Mutually exclusive RAS point mutations or PAX8-PPARγ translocation are reported in 75% of follicular thyroid carcinomas.

b More common in classic PTCs.

RAS gene mutations (NRAS, HRAS, and KRAS) are frequently identified and can be found in both benign and malignant thyroid neoplasms. The ROM associated with each RAS gene mutation is detailed in Table 5.^13^

**Table 5:** Types of RAS mutations and reported risk of malignancy.^12^

HRAS	27%
NRAS	15%
KRAS	14%

### What are the most commonly used commercial molecular testing platforms currently available to supplement thyroid FNA cytology?

Several commercially available molecular tests are used on FNA samples from thyroid nodules to help assess malignancy risk, particularly in indeterminate cases. The most widely used platforms include the Afirma gene expression classifier (GEC)/gene sequencing classifier (GSC), ThyGeNEXT/ThyraMIR, and ThyroSeq. These tests aid in risk stratification and inform management decisions. Table 6 summarizes the collection methods, testing methodology, gene targets, and the advantages, and limitations of each platform.

**Table 6:** Most commonly used commercial thyroid molecular testing platforms.

*Test*	*Collecting methods*	Methodology	Gene targets	Advantages	Disadvantages
***Afirma GEC/GSC***	Fresh FNAC passes into respective nucleic acid preservative.	Machine learning algorithms to analyze RNA sequencing and transcriptome expression.	A panel of 167 genes.	Excellent as a “rule-out” test in Bethesda III and IV lesions. The latest platform (GSC + XA) decreases the rate of surgery in ITN patients by 60%.	Can increase the rate of overtreatment. Since it doesn't offer slide scraping, it can be difficult to perform if it wasn't done at the time of initial FNAC.
***ThyGeNEXT/ThyraMIR***	Slide scraping^a^, Fresh FNAC passes into respective nucleic acid preservative.	Targeted sequencing for DNA, RNA fusions, and miRNA to detect different genetic alterations.	DNA targets: KRAS, NRAS, PIK3CA, BRAF, HRAS, PTEN, GNAS, ALK, TERT promoter region, and RET. RNA targets: RET, THADA, NTRK, PPARy, ALK and BRAF. 38 different fusion transcripts.	Excellent as a “rule-out” test in Bethesda III and IV lesions. Decreases the rate of surgery in ITN patients by 60%.	Post validation studies are limited as this is a relatively new molecular test.
***Thyroseq***	Slide scraping^a^, Fresh FNAC passes into respective nucleic acid preservative, FFPE, cell block.	Targeted sequencing for DNA and RNA.	A panel of 112 genes.	More cost effective than Afirma GSC or lobectomy. Excellent as a “rule-out” test in Bethesda III and IV lesions.	Can increase the overall cost of care of patients with thyroid nodules, especially since it hasn't been shown to decrease the rate of surgery.

ITN: indeterminate thyroid nodule; FNAC: fine needle aspiration cytology; FFPE: formalin-fixed paraffin embedded.

a The use of microscope assisted microdissection to collect thyroid follicular cells from cytology slides.

The Afirma GEC assesses malignancy risk based on messenger RNA (mRNA) expression.^13^

ThyroSeq V3 uses targeted DNA and RNA sequencing to detect a wide range of molecular changes, including gene expression alterations, mutations, copy number variations, insertions/deletions, and gene fusions.

ThyGeNEXT/ThyraMIR is a combined platform that checks gene mutations and fusions for malignancy diagnosis. If ThyGeNEXT results are negative, the ThyraMIR test is reflexed to analyze miRNA gene expression. Results from both tests are integrated for more precise risk stratification. All three platforms- Afirma GC, ThyroSeq V3, and ThyGeNEXT/ThyraMIR have demonstrated comparable sensitivity and specificity in detecting relevant molecular alterations in thyroid nodules.^14^^,^^15^

## Diagnostic findings, Part 7

Final surgical resection (lobectomy) of this thyroid nodule was diagnostic of noninvasive follicular thyroid neoplasm with papillary-like nuclear features (NIFTP) (figure: 1B).

## Questions/discussion points, Part 7

### What are the histologic features of noninvasive follicular thyroid neoplasm with papillary-like nuclear features?

As the name suggests, NIFTP refers to a group of follicular-cell-derived thyroid neoplasms that are encapsulated, exhibit a follicular growth pattern, and show nuclear features similar to PTC. Key histologic criteria for NIFTP diagnosis include a completely encapsulated tumor, a pure follicular growth pattern with no true papillae (except for the rare, limited presence of <1% fibrovascular core), and no capsular or vascular invasion, confirmed by thorough microscopic examination of the entire capsule. In addition, these tumors should also exhibit papillary-like nuclear features, including (1) nuclear size and shape alterations: enlargement, elongation, overlapping, and crowding (2) nuclear membrane irregularities: irregular contours, grooves, and intranuclear pseudoinclusion, and (3) chromatin clearing (giving nuclei an “Orphan Annie eye” appearance). Although the presence of rare fibrovascular cores is permitted, their identification should prompt submission of additional tissue sections to exclude true papillary carcinoma. According to the World Health Organization's 5th edition of Endocrine and Neuroendocrine tumors, the term “carcinoma” is no longer recommended for NIFTP, reflecting its extremely low malignant potential. The preferred terminology is now noninvasive follicular thyroid NIFTP.

### What are the subsequent management approaches for noninvasive follicular thyroid neoplasm with papillary like nuclear features?

Because NIFTP is noninvasive and has a very low risk of recurrence or metastasis, its management is generally conservative. Recommended treatment includes lobectomy, which is sufficient in most cases and preserves thyroid function. Total thyroidectomy may be considered in the presence of additional risk factors (e.g. bilateral nodules), but this approach requires lifelong levothyroxine therapy. Radioactive iodine (RAI) treatment is not recommended, as it is unnecessary for noninvasive lesions. The prognosis is excellent, with virtually no risk of metastasis or recurrence when adequately resected.

## Teaching points

• Follicular adenoma is a benign, encapsulated tumor that has an excellent prognosis and treated with excision.
• Follicular carcinoma is a malignant follicular tumor. It spreads hematogenously, and its prognosis is worse than that of papillary carcinoma.
• Papillary thyroid carcinoma is the most common thyroid carcinoma and is characterized by nuclear grooves, clearing (Orphan Annie eye) and pseudoinclusions. It has a good prognosis.
• Thyroid imaging reporting and data systems scoring is an important component of evaluating the ROM and determining which nodules need further evaluation, for example, FNA, evaluation for malignancy, and additional treatment.
• Fine-needle aspiration cytology is an essential tool for evaluating suspicious thyroid nodules.
• Fine-needle aspiration cytology results are reported using the Bethesda System for Reporting Thyroid Cytopathology (TBSRTC), recently updated in 2023.
• Molecular testing can be used to further assess the ROM in nodules with indeterminate cytology (Bethesda categories III-V). Alternatively, surgical resection can be performed based on clinical findings or patient preference.
• Common molecular testing platforms used with FNA samples include the Afirma gene expression classifier (GEC)/gene sequencing classifier (GSC), ThyGeNEXT/ThyraMIR, and ThyroSeq. These platforms have shown comparable sensitivity and specificity in detecting mutations in thyroid nodules.
• Standard management of NIFTP is lobectomy without the need for radioactive iodine therapy.

## Funding

The article processing fee for this article was funded by an Open Access Award given by the Society of ‘67, which supports the mission of the Association for Academic Pathology to produce the next generation of outstanding investigators and educational scholars in the field of pathology. This award helps to promote the publication of high-quality original scholarship in *Academic Pathology* by authors at an early stage of academic development.
